# Experimental Study of the Post-Fire Mechanical and Material Response of Cold-Worked Austenitic Stainless Steel Reinforcing Bar

**DOI:** 10.3390/ma15041564

**Published:** 2022-02-19

**Authors:** Fazal-ur Rehman, Katherine A. Cashell, Lorna Anguilano

**Affiliations:** 1 Department of Civil and Environmental Engineering, College of Engineering, Design and Physical Science, Brunel University London, Kingston Lane, London UB8 3PH, UK; katherine.cashell@brunel.ac.uk; 2 Experimental Techniques Centre, College of Engineering, Design and Physical Science, Brunel University London, Kingston Lane, London UB8 3PH, UK; lorna.anguilano@brunel.ac.uk

**Keywords:** stainless steel, residual properties, fire, post-fire, austenitic, cold-worked, tensile test, metallurgical assessment, material characterization

## Abstract

This paper is concerned with the behaviour of stainless steel reinforcing bar following exposure to elevated temperatures from a fire, followed by subsequent cooling. Stainless steel-reinforced concrete is an increasingly popular solution for structural applications which require corrosion resistance, excellent mechanical properties, and long life cycles with little maintenance. In addition, although stainless steel reinforcement has a higher initial cost compared with traditional carbon steel bars, the overall life cycle costs are likely to be quite similar, owing to the lack of maintenance required for stainless steel materials. There is no information available in the literature on the post-fire properties of austenitic stainless steel reinforcement, although these data are essential for any engineer who wishes to study the structural integrity of a reinforced concrete component or system following a fire. Accordingly, this paper presents a detailed discussion and analysis from the results of a series of laboratory experiments on three grades of austenitic stainless steel reinforcement following various levels of temperature exposure and also different cooling rates. Both the mechanical and metallurgical properties are examined, and the behaviour is compared to that of B500B carbon steel reinforcement. It is shown that the stainless steel bars retained their mechanical properties under the majority of the scenarios examined and to a greater degree than traditional materials. This is important for the rehabilitation and salvage of existing reinforced concrete structures following a fire and also to avoid unnecessary demolition and replacement.

## 1. Introduction

This paper is concerned with the post-fire behaviour of austenitic stainless steel reinforcing bar and examines both the metallurgical and mechanical properties which affect its performance. Stainless steels are alloys of iron containing at least 10.5% chromium and up to 0.07% carbon. The austenitic grades are a subset of stainless steels which contain stabilizing elements, such as nickel and manganese [1]. The use of austenitic stainless steels in structural applications has increased in recent years, as more design information has become available, and greater cost-effectiveness has been achieved. Much of the attention from the research community, however, has focussed on bare stainless steel structural sections, such as I-sections and tubular members, with less focus given to stainless steel reinforcement for concrete. Nevertheless, stainless steel reinforcement is an increasingly popular solution for structures susceptible to corrosion and can result in lower maintenance costs and higher life spans (>125 years) in comparison with traditional carbon steel-reinforced concrete. However, unlike carbon steel rebar, the understanding of how austenitic stainless steel reinforcement performs both during and following a fire scenario is very much unknown, owing to an absence of performance data in the available literature.

The post-fire behaviour of building structures and infrastructure is important for several reasons. First, having an immediate, realistic understanding of the residual strength and stiffness remaining in the structure following exposure to fire allows emergency response teams, fire fighters and investigators to make informed decisions about rescue and salvage operations, as well as the risk of collapse. In addition, this enables informed decisions to be made regarding the rehabilitation and repurposing of the structure. Failure to understand the post-fire response and capacity of structural materials and elements may result in unnecessary demolitions and loss of structures, which is neither environmentally nor economically sustainable.

It is noteworthy that the production route, including whether the bars have been hot-rolled or cold-worked during the production process, is very influential on their mechanical properties. Stainless steel reinforcement that has been cold-worked is significantly harder than hot-rolled bars, resulting in higher tensile and yield strengths. On the other hand, hot-rolled stainless reinforcing steel is generally more ductile than cold-worked material. The production of stainless steel reinforcement is also different from stainless steel plate in that it is rarely solution-annealed following hot-rolling and cold-working. These differences may lead to variations in the mechanical properties in normal conditions, as well as during and following a fire.

Existing information and previous studies on the post-fire performance of austenitic stainless steel rebars are limited, and the most relevant works are detailed in Table 1. Felicetti et al. [2] studied the mechanical response of rebars made from grade 1.4307 austenitic stainless steel, exploring both cold-worked and hot rolled bars. All of the specimens examined had a ribbed profile and were annealed. Zeng et al. [3] presented some analysis on the post-fire response of grade 1.4401 rebars, although limited details of the rebar specifications were provided. (It is not known if the specimens were hot-rolled or cold worked, for example.) In both of these experimental programs, the rebar specimens were heated to a target temperature between 200 and 900 °C, held at that temperature for a pre-determined period of soaking time, and then cooled to room temperature before a tensile test was conducted to obtain the post-fire mechanical properties. Zeng et al. [3] also included an analysis of the corrosion resistance of the rebars.

The tensile test data from both programmes show that stainless steel rebar is reusable following exposure to up to 700 °C, but excessive plastic elongation occurs following exposure to higher temperatures. The influence of the production route of the reinforcing bars was assessed by Felicetti et al. [2], who studied both cold-worked and hot-rolled bars. It was shown that there is little difference between the original and post-fire behaviour for the hot-rolled rebars after heating to up to 900 °C. On the other hand, for the cold-worked samples, there was a significant disparity between the original properties and those following exposure to fire and subsequent cooling. It was shown that the cold-worked samples heated to above 400 °C exhibited a significant increase in the ultimate strain in the post-fire mechanical properties. Interestingly, Zeng et al. [3] also compared the response of bars that were heated for various time periods and then subsequently cooled, and it was shown that the post-fire properties were very similar regardless of whether the bars were exposed to elevated temperatures for 1 h or 7 days.

In order to understand the post-fire mechanical behaviour of stainless steel reinforcement, it is important to understand the metallurgy and specifically the effects of the relevant phases of the microstructure (i.e., the austenite, ferrite, and martensite phases). For stainless steel, the parent element is iron (Fe), which is ferritic at ambient temperatures and is also allotropic by nature, meaning that as the temperature rises, the ferrite phase transitions into an austenite phase. With the addition of various austenite-promoting elements such as nickel and manganese, this austenite phase can be stabilised at an ambient temperature, creating an austenitic alloy. The microstructure of the austenitic alloy can be further influenced through mechanical measures, and cold working is the usual approach adopted to increase strength, as austenitic stainless steels possess a high strain hardening rate. When grade 1.4301 stainless steels are cold-worked, the γ-austenite grains can undergo a stress-induced transformation into ε-martensite and then α′-martensite, or they may experience a strain-induced transformation, resulting in twinning and then the formation of α′-martensite. For both scenarios, there is a formation of α′-martensite, as the chemical composition does not change, and the alloy remains austenitic.

In this context, the current paper presents a mechanical and metallurgical assessment of the behaviour of austenitic stainless steel rebars following exposure to various levels of elevated temperatures and subsequent cooling. The aim is to provide performance information and analysis which is useful for engineers to assess the post-fire behaviour of stainless steel-reinforced concrete elements, to promote the re-use and rehabilitation of structures post-fire, and to avoid unnecessary structural loss or demolitions following a fire. An extensive experimental programme is described, in which different grades of stainless steel with various diameters are subjected to a range of heating and cooling patterns. The results are compared to the behaviour of traditional carbon steel reinforcement to enable a direct assessment of how the stainless steel reinforcement performs relative to the traditional materials.

The test programme comprised both mechanical tensile tests as well as a metallurgical examination to give a full overview of the behaviour and insight into the inter-relationship between the metallurgy and the mechanical behaviour following exposure to elevated temperatures. The paper proceeds with a detailed description of the test programme, including the material selection and preparation, mechanical testing regime, and metallurgical assessment. This is followed by a discussion of the findings and concluding remarks.

## 2. Experimental Investigation

A total of 204 samples were examined in this programme, including 120 mechanical tests which were performed in the civil engineering laboratory at Brunel University London and 84 metallurgical tests which were conducted in the Experimental Techniques Centre (ETC), also at Brunel. The aim of the test programme was to understand the post-fire properties and behaviour of austenitic stainless steel rebar, and as such, the samples were all heated to a target temperature and then cooled before testing.

### 2.1. Material Selection and Preparation

A key aim of this work was to study a material which was commonly used by engineers in practice and likely to be available from a supplier. Accordingly, the reinforcement was selected based on the available stock at local suppliers in the UK. Three different types of ribbed, cold-worked stainless steel reinforcement and one carbon steel rebar were included in the test programme, and the composition of each is given in Table 2. The data in the table were provided by the suppliers, whilst the limits are given in BS EN 10088-1:2014 [1]. The materials examined include the following:

(i) Grade 1.4301 (in accordance with BS EN 10088, but also known as grade 304) with a diameter d of 8 mm: the cheapest and most commonly available chromium-nickel austenitic stainless steel reinforcement, which offers moderate corrosion resistance as well as good strength, stiffness, and durability and excellent ductility.
(ii) Grade 1.4401 (also known as grade 316) with a diameter d of 12 mm: a chromium-nickel-molybdenum stainless steel with very good corrosion resistance (similar level to the duplex grades), owing to the high molybdenum content, which also makes it more expensive than grade 1.4301 in terms of initial costs. Therefore, it is commonly employed in harsh environments such as marine or industrial settings. In terms of mechanical properties, it offers characteristics which are comparable to carbon steel in terms of strength, ductility, and stiffness.
(iii) Grade 1.4436 (also known as grade 316L) with a diameter d of 8 mm: a very low-carbon stainless steel, designated by the “L”, which is otherwise similar to grade 1.4401 as described above. Grade 1.4436 has improved resistance to intergranular corrosion in the heat-affected zone (HAZ) of welds.
(iv) Grade B500B carbon steel with a diameter d of 8 mm: a common carbon steel rebar, offering moderate tensile properties but poor corrosion resistance. Carbon steel rebar is cheap to manufacture due to the lack of precious elements used in the alloy and more readily available than stainless steel, making it ideal to use in structures that are at low risk of corrosion.

**Figure 1:**
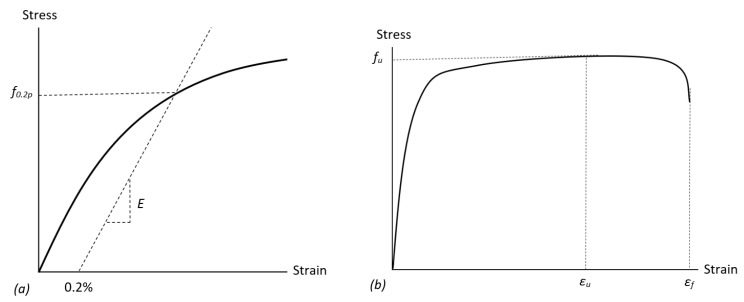
Idealised stress–strain response of austenitic stainless steel reinforcement, including (**a**) a focused view of the initial part of the curve and (**b**) the complete behaviour.

There are no specific requirements for the manufacturing processes of stainless steel rebars, but BS 6744-10:2016 [4] provides requirements for the chemical composition and also the testing methods for stainless steel bars used for the reinforcement of concrete. In accordance with this standard, the minimum required mechanical property values of stainless steel rebar are based on the carbon steel B500B specifications as given in Part 7 of BS 4449:2005 [5]. BS 6744-11:2016 [4] also states that the stainless steel rebar is required to have a minimum total strain at failure *ε_f_* of at least 14%, as opposed to the 5% specified in BS 4449:2005 [5] for carbon steel. For clarity on the terms used in this paper, Figure 1 presents an idealisation of the stress–strain response for stainless steel reinforcement, indicating the key characteristics.

### 2.2. Heating and Cooling of the Specimens

The samples were heated to a target temperature of between 100 °C and 900 °C in a Carbolite CWF 1100 furnace at a rate of 10 °C/min using a Eurotherm 3216 control module to monitor the temperature in the chamber. The work performed by Twilt (1998) found that when heating steel, a heating rate of between 5 and 50 °C/min may be considered realistic, with 10 °C considered to be a reasonable rate for a regular fire [6]. Although this work was focused on structural steel sections, it gives an indication of how the temperature spreads through steel during a fire. The temperature was then kept constant at the target temperature for a soaking period of 1 h. The highest temperature examined was 900 °C, and this was considered to be the upper bound for the temperature that rebar is likely to reach in a real fire. In this scenario, it is likely that large deflections will occur, along with excessive cracking of the concrete, and therefore, the rebars can reach very high temperatures. The work performed by Venanzi et al. (2008) documented actual fire scenarios where rebar was left exposed in a reinforced concrete structure following a fire, illustrating the combined effects of the elevated temperature exposure and the force from water jets used by the fire brigade [7].

**Figure 2:**
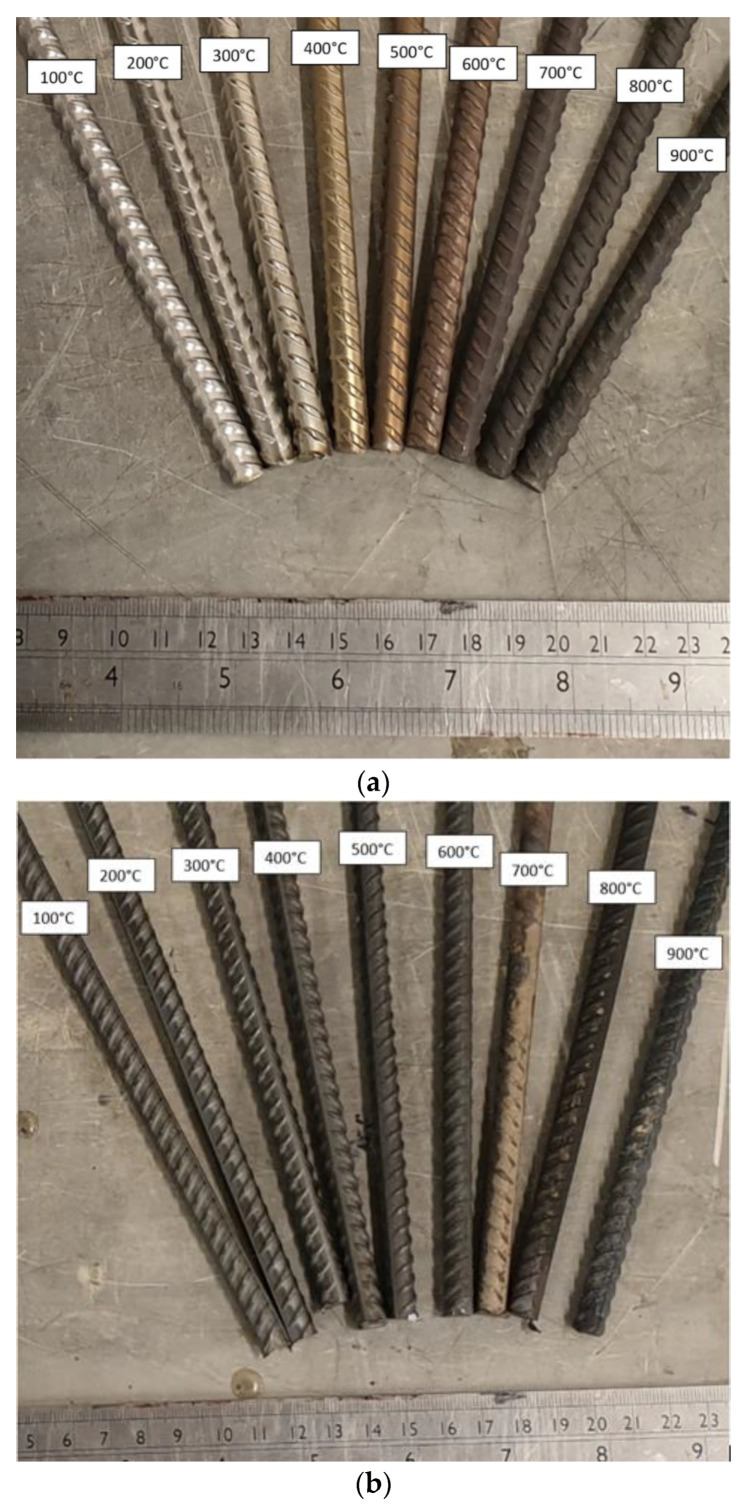
(**a**) Grade 1.4301 stainless steel and (**b**) grade B500B carbon steel samples following exposure to elevated temperatures and subsequent cooling.

Three different cooling methods were employed in the test programme for the heated samples, including (1) quenching in cold water, (2) slow, controlled cooling in the furnace at a rate of 1 °C/min, and (3) cooling naturally in air to room temperature. Moreover, three repetitions were conducted for every grade of rebar tested per temperature point. The three cooling methods were selected to give the full range of realistic cooling rates following a fire scenario, such as the fire going out naturally or the structure being subjected to water by the fire brigade, and to establish if this is an influential factor on the residual properties. It is noteworthy that in the current paper, the phrase “residual” when employed with a mechanical property refers to the remaining value of that property following exposure to fire and then subsequent cooling. Figure 2 shows the surface state of (a) grade 1.4301 stainless steel rebars following exposure to various levels of elevated temperatures and subsequent cooling by quenching the bars in cold water, as well as (b) the corresponding images for the carbon steel rebars. It is clear that there were increasing levels of surface discoloration following exposure to higher temperatures.

### 2.3. Mechanical Testing Arrangement

**Figure 3:**
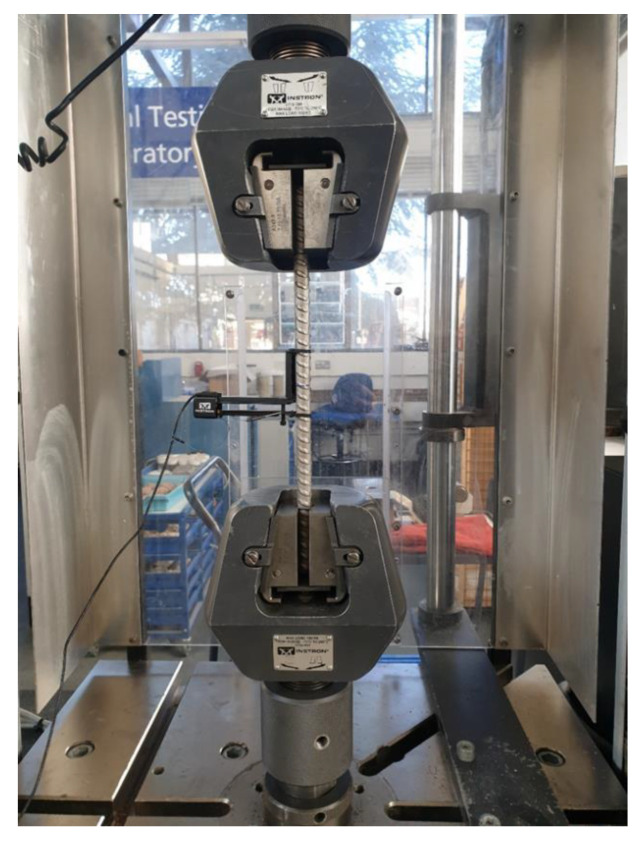
Set-up for tensile testing on Instron 5584.

The ambient temperature tensile testing was conducted using an Instron 5584 electromechanical testing frame with a load capacity of 150 kN. The strain measurements were recorded using an Instron EX2620-601 extensometer as shown in Figure 3, which recorded up to around 10% total strain. Higher strains were measured by the testing machine, and these values were corroborated using the extensometer data. In addition, in order to obtain a direct and reliable measurement after cooling of the ultimate mechanical strain, the rebars were marked clearly at 10-mm intervals before testing, and then the final strain post testing was determined by carefully placing the bars back together and measuring the total length between the adjacent markings at the location of the break. The tensile tests were conducted in accordance with the guidance given in BS EN ISO 6892-1:2016 [8], and the samples had a total length (L_t_) of 300 mm, a gauge length (L_o_) of 50 mm, and a parallel length (L_c_) between the gripping jaws of 200 mm. A 10-kN preload was first applied to each specimen at a rate of 0.05 mm/min. Then, an initial strain rate of 0.00007 s^−1^ was applied until a strain value of 1% was reached, followed by increasing the strain rate to 0.00025 s^−1^ over 5 min. This strain rate was maintained until fracturing of the rebar occurred.

### 2.4. Metallurgical Characterization

As stated before, a key aim of this study is to understand the relationship between the mechanical performance of stainless steel rebars and their evolving metallurgical characteristics following exposure to various levels of elevated temperatures and subsequent cooling. Accordingly, in this part of the investigation, the samples were initially inspected at each level of elevated temperature exposure and subsequent cooling method through a phase identification analysis. Then, the samples with the most notable changes underwent further examination through a grain inspection. X-ray diffraction was used as the preliminary method for the phase identification analysis, and this was carried out using a Bruker D8 Advance diffractometer equipped with a copper source and Lynxeye position sensitive detector. For the grain image collection, backscatter detector imaging was used on a Zeiss Crossbeam, and the image analysis was conducted using Fiji ImageJ. The purpose of these tests was to establish a complete scenario of any phase changes that may have occurred during the heating and cooling process and also to inspect the grain profile for the most prominent samples to determine whether different cooling patterns had a notable effect.

## 3. Mechanical Test Results

A total of 120 tensile tests were conducted on samples that were heated to a defined target temperature of between 100 and 900 °C and then subsequently cooled by one of the three methods previously described. A total of three repeats were conducted on each scenario. and the average results are presented. It is noteworthy that very little variation was observed in the results between the repeat samples. The results are presented alongside the graphical data in Table 3, Table 4 and Table 5 for the austenitic stainless steel samples and Table 6 for the carbon steel rebars. A reference system was adopted to label each specimen as follows: the first parameter denotes the material grade (either 1.4301, 1.4401, or 1.4436 for stainless steel and B500B for carbon steel), next is the target elevated temperature, whilst the final parameter describes the cooling method used, where Q is for quenched in water, A is for air cooling, and S is for slow cooling. The measured data from the tests included the 0.2% proof strength *f*_0.2*p*_, the tensile strength *f_u_*, the percentage total elongation at the maximum force *ε_u_*, the total strain at failure *ε_f_*, and the modulus of elasticity *E*. Each of the grades examined are discussed in more detail in the following subsections.

### 3.1. Grade 1.4301

**Figure 4:**
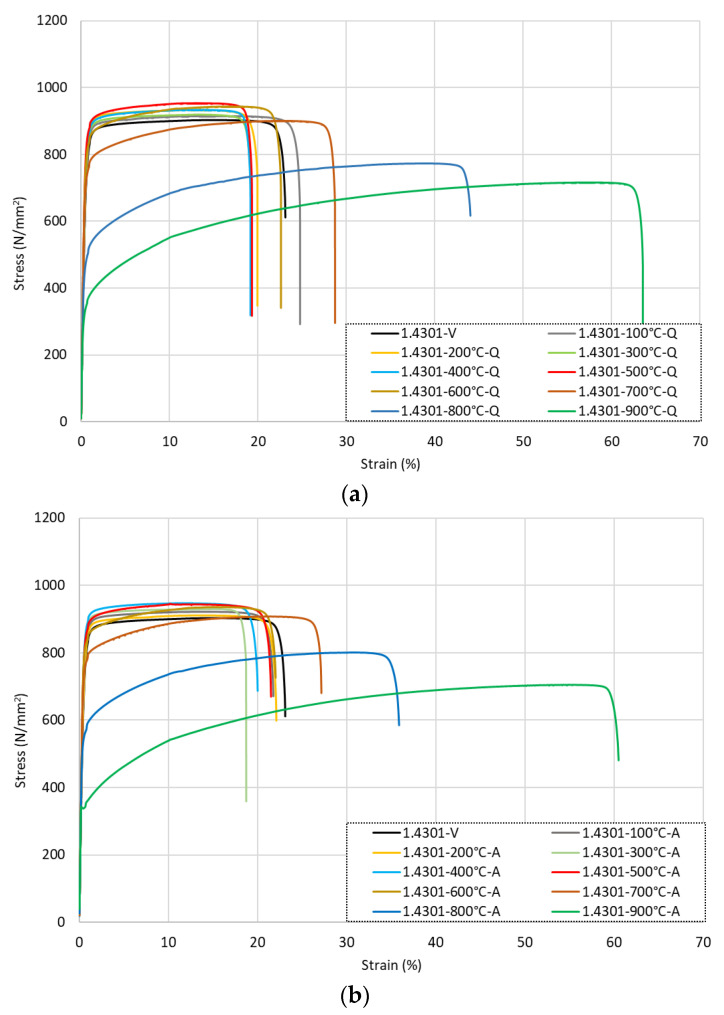

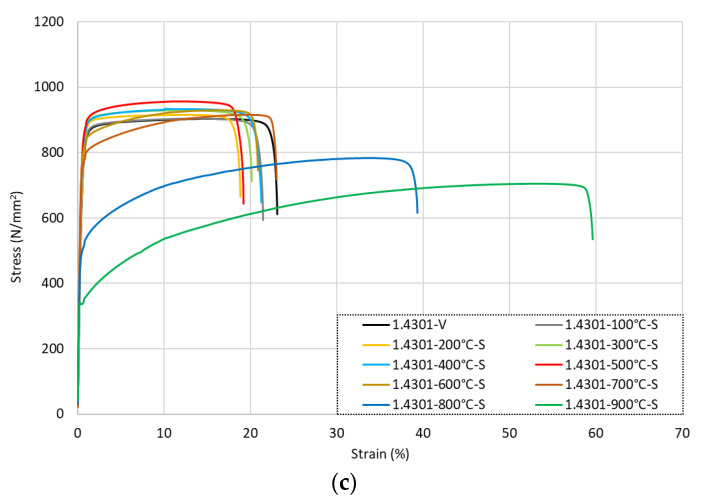
Stress–strain responses for grade 1.4301 stainless steel rebars following exposure to elevated temperatures and then (**a**) quenched in water, (**b**) cooled naturally in air, or (**c**) slow-cooled in the furnace.

The full stress–strain responses for the grade 1.4301 samples following exposure to various levels of elevated temperature and then cooling are presented in Figure 4 for (a) samples that were cooled by quenching in water, (b) rebars that were air-cooled, and (c) the slow-cooled bars. It is clear that following low-to-moderate levels of heat exposure up to around 500 °C, the strength was completely retained and even marginally increased in some cases compared with the virgin unheated samples. In addition, the ductility remained relatively unchanged.

With reference to the data presented in Table 3, it is shown that there was a 2–7% increase in the *f*_0.2*p*_ value for every 100 °C increment from the virgin (ambient) sample until the specimens that were exposed to 500 °C. The total increase in *f*_0.2*p*_ over this temperature exposure range was 15%, and this value remained consistent regardless of the cooling regime. The increase was slightly lower for the *f_u_* value, although a similar trend was observed; this property increased by around 5% following exposure to 500 °C compared with the corresponding virgin sample. On the other hand, for the strain response, the *ε_u_* value was relatively unchanged, remaining within 1% of the virgin sample value, whilst the total strain at failure *ε_f_* generally showed a reduction in value for the samples that were heated up to 500 °C before cooling of between 5%–20%. It is noteworthy that one sample that was quenched after being heated to 100 °C had a 6% increase in *ε_f_*, but all other samples had a reduction in the ultimate strain.

After higher levels of elevated temperature exposure of between 600 °C and 700 °C, both the *f*_0.2*p*_ and *f_u_* values almost fully returned to the corresponding virgin values. For the 600 °C sample, the *ε_u_* values increased by 56–67%, whilst the *ε_f_* values rose in comparison with the low-to-moderate specimens but still underperformed against the virgin sample. Following exposure to 700 °C, this trend continued with an increase in *ε_u_* by a further 41–60% and *ε_f_* by 38–69%. This different strain behaviour resulted in a more rounded stress–strain response, as shown in Figure 4. This was most likely because the effects of cold working on the material were lost after exposure to higher temperatures and were not regained following cooling. After exposure to higher temperatures of 800 °C and 900 °C, there was a notable loss in strength for the samples, with *f*_0.2*p*_ reducing by 25–36% for the 800 °C samples compared with the room temperature behaviour and 53–56% for the 900 °C specimens. There was a similar trend for *f_u_*, although the losses were less significant, with a 12–15% reduction for the bars heated to 800 °C and a 21–23% reduction for the 900 °C specimens. On the other hand, the strain values were much higher than the equivalent virgin sample values, as *ε_u_* increased by between 206 and 241% following exposure to 800 °C compared with the virgin values and between 441 and 483% for the 900 °C samples. For the total elongation, *ε_f_* again showed a similar trend, although to a lesser extent compared with *ε_u_*, with a 54–89% increase for the 800 °C bars and 156–172% increase for the 900 °C samples compared with the virgin samples. The modulus of elasticity *E* showed very little change following exposure to elevated temperatures and subsequent cooling, with the lowest and highest values recorded across all tests being 181.9 and 197.7 GPa, respectively. These values were within 10% of the guideline value set out in BS 6744 of 190 GPa for austenitic stainless steels.

**Figure 5:**
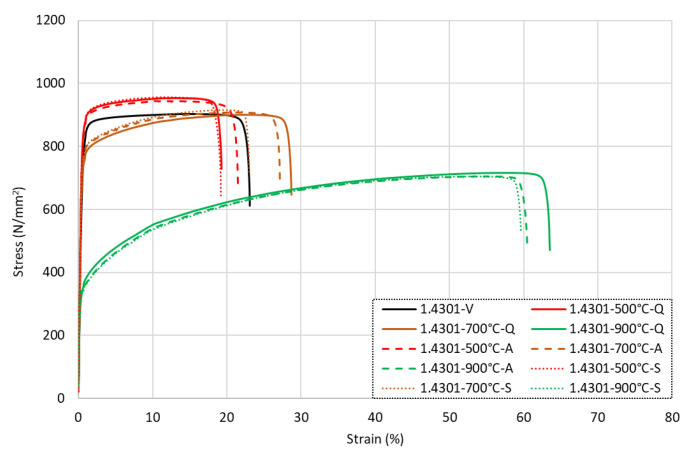
Stress–strain responses for grade 1.4301 stainless steel rebars following exposure to 500 °C, 700 °C, and 900 °C and various cooling.

In order to conduct a closer inspection of the influence of the cooling method on the post-fire behaviour, Figure 5 presents the grade 1.4301 rebars that were heated to either 500 °C, 700 °C, or 900 °C and then cooled by quenching in water, air-cooling, or slow-cooling in the furnace. The data for the unheated virgin specimen are also included. In this image, the solid lines are for the quenched samples, the dashed lines are for the air-cooled rebars, and the dotted lines are for the slow-cooled specimens. It is evident that for these samples, the cooling method did not have a strong influence on the post-fire behaviour. For the bars exposed to higher levels of elevated temperatures, the quenched specimens demonstrated greater ductility compared with the samples cooled more slowly, but the strength remained similar. For all levels of temperature exposure, the slow-cooled specimens had the lowest level of ductility following heating and cooling compared with the samples that were cooled either by quenching in water or naturally cooling in air.

### 3.2. Grade 1.4401

**Figure 6:**
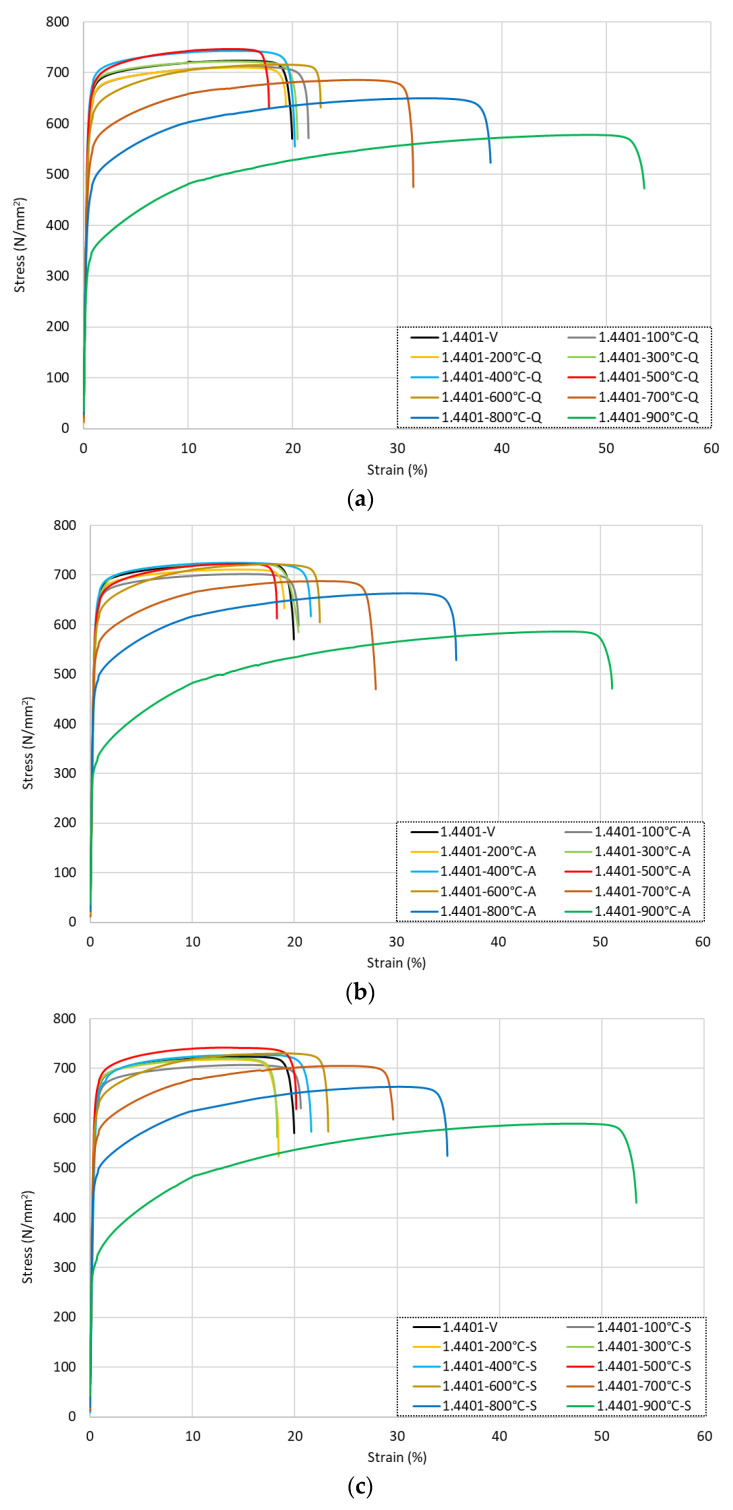
Stress–strain responses for grade 1.4401 stainless steel rebars following exposure to elevated temperatures and then (**a**) quenching in water, (**b**) cooling naturally in air, or (**c**) slow-cooling in the furnace.

Figure 6 presents the full stress–strain response for the grade 1.4401 samples that were heated to different levels of elevated temperatures and then cooled either by (a) quenching in water, (b) air-cooling, or (c) slow-cooling in the furnace. It is observed that for the samples that were heated up to around 500 °C, there was very little change in the strength or ductility following cooling by any method. In this range, for the samples that were heated up to 500 °C, the data presented in Table 4 show that *f*_0.2*p*_ and *f_u_* increased by between 1 and 6% and 1 and 3%, respectively, compared with the virgin samples. For the strain response in this same temperature exposure range, both *ε_u_* and *ε_f_* increased by between 1 and 10% compared with the virgin sample values.

Generally, the samples retained (or lost and regained) their strength and ductility once they returned to room temperature following elevated temperature exposure. For the specimens that were heated to 600 °C or higher, there was a clearer change in the overall behaviour, with a more rounded response observed and a slight loss in strength accompanied by an increase in the ultimate strain following cooling. Following exposure to 600 °C, the strength performance remained consistent with those at lower temperatures, whilst the strain response showed an increase for *ε_u_* and *ε_f_* of 19–25% and 13–17% compared with the original values. Following exposure to higher temperatures, the loss in strength became more pronounced, as did the increase in ductility. After heating to 700 °C, *f*_0.2*p*_ and *f_u_* reduced by 10–17% and 3–5%, respectively, against the virgin values, whereas *ε_u_* increased by 54–75% and *ε_f_* rose by 40–58% in these same tests. Following heating to higher temperatures of 800 °C and 900 °C, *f*_0.2*p*_ reduced by 23–30% and 49–51%, respectively, whereas *f_u_* reduced by only 8–10% (800 °C) and 19–20% (900 °C). There were very significant changes in the strains at the ultimate load and failure, as *ε_u_* and *ε_f_* increased by 105–124% and 75–95%, respectively, after exposure to 800 °C and 218–232% and 156–169% for the samples heated to 900 °C. As with the data from grade 1.4301, this illustrates that the cold-working effect was regained in the samples that were heated to approximately 500–600 °C and then cooled, whereas it was permanently lost following exposure to higher levels of elevated temperatures.

This phenomenon was further evidenced by a closer inspection of the total strain at failure *ε_f_*, as this property was largely unchanged compared with the virgin sample following exposure to 500 °C and subsequent cooling, whilst the bars that were heated to higher temperatures had greater failure strains, albeit with a significant reduction in strength. As with grade 1.4301, there was little variation in the elastic modulus values, with the minimum *E* across all tests being 180.6 GPa and the corresponding maximum values being 198.2 GPa, both of which are within 10% of the guideline value of 190 GPa in BS 6744:2016 [5].

**Figure 7:**
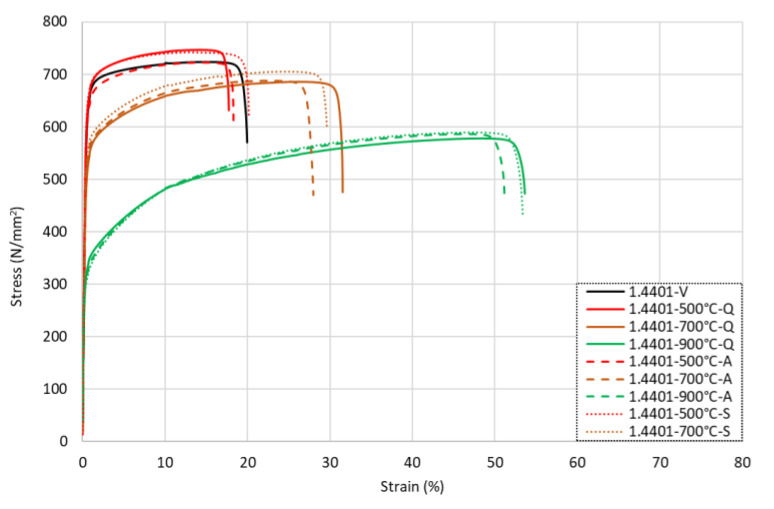
Stress–strain responses for grade 1.4401 stainless steel rebars following exposure to 500 °C, 700 °C, and 900 °C with various cooling methods.

The influence of the cooling method on the stress–strain response of the grade 1.4401 stainless steel rebars following exposure to either 500, 700 °C, or 900 °C is illustrated in Figure 7. Overall, as with grade 1.4301, there was a relatively small variation in the behaviour between the samples that were cooled by different methods. In contrast to grade 1.4301, for all levels of temperature exposure, the grade 1.4401 bars that were air-cooled had the lowest level of ductility, whilst the bars that were exposed to moderate-to-high temperatures and then cooled quickly by quenching in water had slightly lower strengths than the other methods.

### 3.3. Grade 1.4436

**Figure 8:**
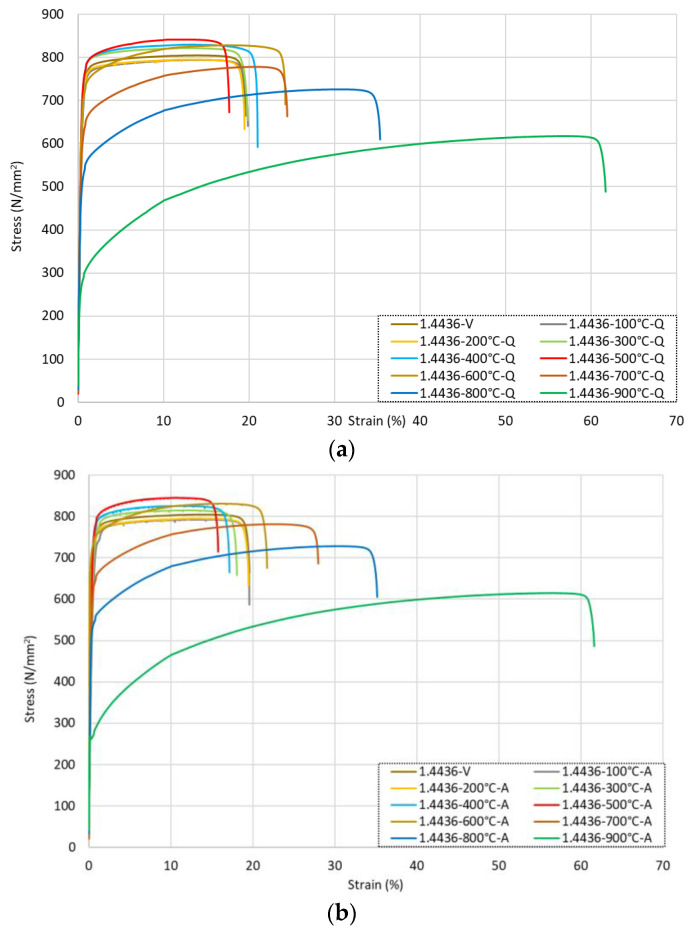

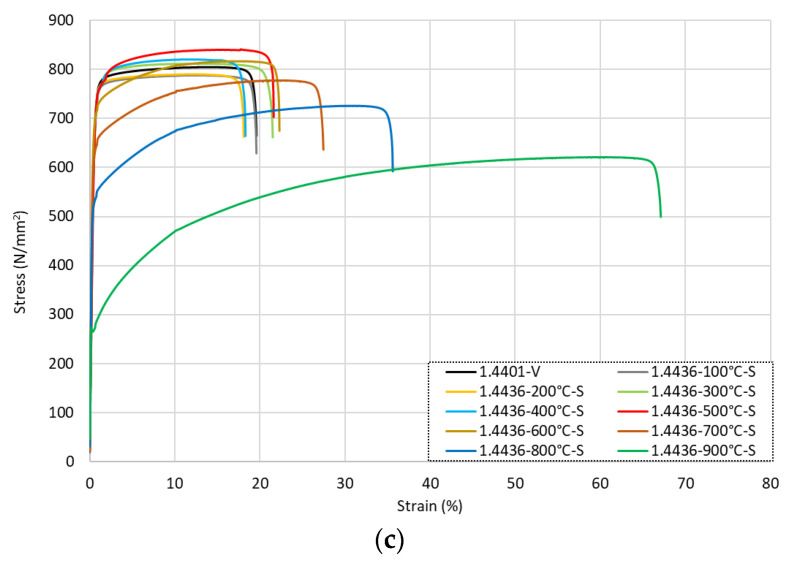
Stress–strain responses for grade 1.4436 stainless steel rebars following exposure to elevated temperatures and then being (**a**) quenched in water, (**b**) cooled naturally in air, or (**c**) slow-cooled in the furnace.

The full stress–strain responses from all grade 1.4436 samples are presented in Figure 8 for (a) the bars quenched in water, (b) the air-cooled samples, and (c) the slow-cooled specimens. It is noted that following exposure to low-to-moderate temperature conditions up to 500 °C, there was a steady rise in the tensile strengths of the samples. There was a relatively slow increase in *f*_0.2*p*_ of between 1 and 5% per 100 °C increment and accumulating to an increase of approximately 11% after heating to 500 °C. The total strength *f_u_* followed a similar trend and increased by around 5% following heating to 500 °C compared with the virgin samples. In this same temperature range (samples that were heated up to 500 °C), *ε_u_* had a negligible change following heat exposure regardless of the cooling method, whilst *ε_f_* increased by around 19%. When comparing the numerical data presented in Table 5 with the stress–strain responses given in Figure 8, it is evident that this variation in strain was not owing to a change in shape of the response as this remained the same, meaning that the material did not undergo any major transformations in grain or phase in this range.

After exposure to higher temperatures ranging from 600 °C upwards, the overall shape of the residual stress–strain response changed as shown in Figure 8 and became more nonlinear. Moreover, the tensile strength gradually declined. Following heating to 600 °C, compared with the virgin sample, there was an increase in strength of between 2 and 6% for *f*_0.2*p*_ and 1 and 3% for *f_u_*, whilst these values for the specimens heated to 700 °C presented a loss of 7–11% for *f*_0.2*p*_ and 3% for *f_u_*. The strain response showed an increase in both *ε_u_* and *ε_f_* of 61–73% and 14–24%, respectively, for the samples heated to 600 °C and 103–128% and 80–82%, respectively, for those heated to 700 °C. After this, for the bars exposed to higher temperatures, there was a significant reduction in the residual strength once the specimen had cooled, with a loss of 21–26% for *f*_0.2*p*_ and 10% for *f_u_* at 800 °C and a loss of 61% for *f*_0.2*p*_ and 23–24% for *f_u_* at 900 °C. There was an increase in *ε_u_* of 206–210% for the bars heated to 800 °C and 476–515% for those that were exposed to a 900 °C temperature compared with the virgin values, as well as corresponding increases in *ε_f_* of 80–82% (800 °C) and 215–343% (900 °C). This resulted in greater roundedness of the overall response, as shown in Figure 8. The modulus of elasticity *E* was observed to show little change, as with the previous austenitic grades.

**Figure 9:**
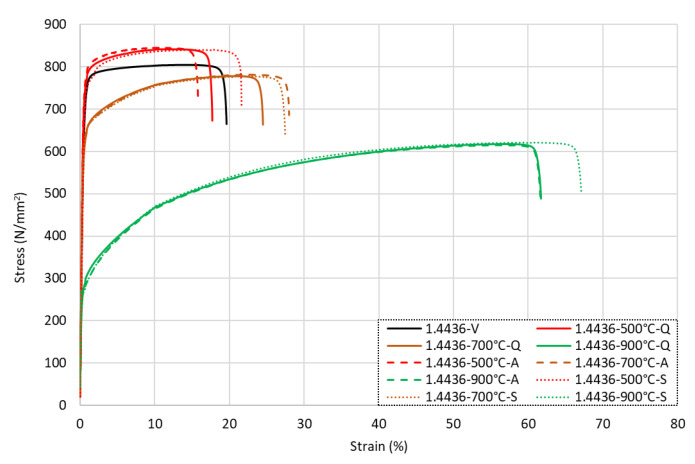
Stress–strain responses for grade 1.4436 stainless steel rebars following exposure to 500 °C, 700 °C, and 900 °C with various cooling.

The influence of the three cooling methods for the grade 1.4436 rebars that were exposed to temperatures of 500 °C, 700 °C, and 900 °C is presented in Figure 9 together with the data for the virgin specimen. As for the other austenitic stainless steel grades discussed before, there were relatively minor differences in the residual response for grade 1.4436 to the different cooling methods. The samples heated to 500 °C showed the greatest variance from the various cooling methods, with the total strain *ε_f_* showing a drop of 19% with the air-cooled sample and an increase of 10% with the slow-cooled sample.

### 3.4. Grade B500B Carbon Steel Rebar

**Figure 10:**
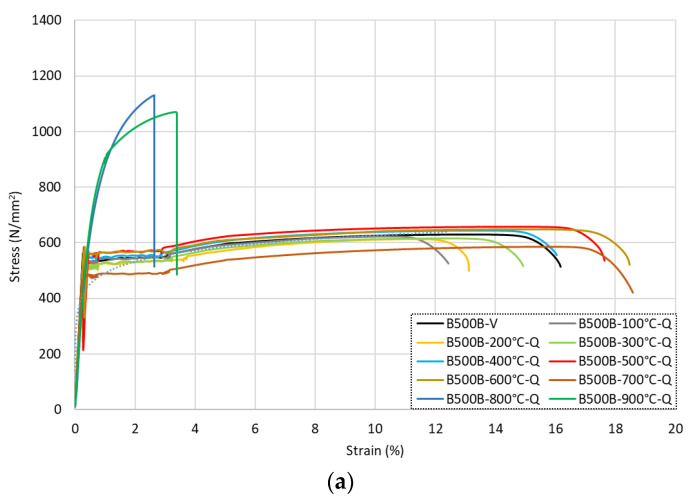

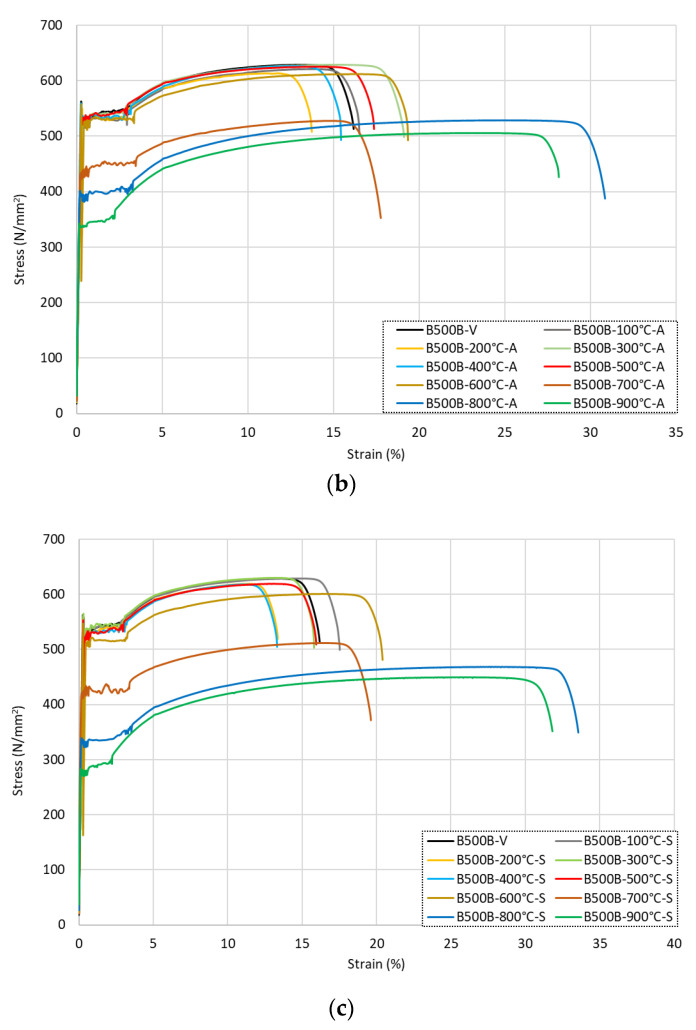
Stress–strain responses for B500B carbon steel rebars following exposure to elevated temperatures and then being (**a**) quenched in water, (**b**) cooled naturally in air, and (**c**) slow-cooled in the furnace.

The full stress–strain responses for the B500B carbon steel reinforcing bars following exposure to various levels of elevated temperatures and subsequent cooling methods are shown in Figure 10 for (a) the quenched samples, (b) the air-cooled samples, and (c) the slow-cooled rebar samples. In addition, the data from the tests are given in Table 6. It is noteworthy that these bars were cold-worked during the production process. The first observation from the results is that, as expected, the overall shapes of the stress–strain curves were quite different to those for the stainless steel bars. In most cases, the carbon steel samples developed a clear yield point, followed by a yield plateau and then a small degree of strain hardening with less overall ductility than the stainless steel samples. On the other hand, the stainless steel specimens had a more rounded, continuous constitutive response.

With reference to the data in Table 6, there are a number of interesting observations on the behaviour. First, for the quenched samples, following exposure to relatively low elevated temperatures (up to 300 °C), *f*_0.2*p*_ and *f_u_* reduced by 0–3% after cooling. On the other hand, for the samples that were cooled more slowly using the other methods, these corresponding values were almost identical to the virgin values, with no significant difference between the residual and virgin values observed. For all cooling methods, there was no notable difference in the residual strain values following exposure to up to 300 °C compared with the original values. Following exposure to higher temperatures (from 400 °C to 600 °C), the residual values *f*_0.2*p*_ and *f_u_* increased by up to 7% and 5%, respectively, after quenching in water. The air-cooled and slow-cooled samples at 400 °C and 500 °C continued to retain *f*_0.2*p*_ and *f_u_* values almost identical to the virgin specimen, while at 600 °C, the *f*_0.2*p*_ and *f_u_* values showed a loss of 1% and 3–4%, respectively. The residual strain values also showed an increase compared with the corresponding virgin values. These increases were between 17 and 32% for *ε_u_* and 14 and 26% for *ε_f_*. It is noted that the increase in the residual strains was inversely related to the speed of cooling, and therefore, the greatest increase in residual *ε_u_* and *ε_f_* values occurred for the specimens that were cooled the slowest. This pattern was much clearer compared with the stainless steel specimens, which had a more varied response to the heating and cooling cycle. This is discussed in more detail later with the metallurgical analysis.

For the bars that were heated to higher temperatures, there were more significant changes to the residual properties. Following exposure to 700 °C, the residual *f*_0.2*p*_ and *f_u_* values reduced by 9–20% and 7–19%, respectively, which were significantly greater reductions than those observed for the austenitic stainless steel samples. The corresponding residual strain values *ε_u_* and *ε_f_* increased by 12–27% and 10–21%, respectively, compared with the virgin values. Following exposure to higher temperatures of 800 °C and above, there were clear changes in the residual behaviour, and these were more dependent on the cooling method compared with the other samples examined. With reference to Figure 10a, the bars that were cooled quickly by quenching in cool water had a significant change in their stress–strain response following exposure to temperatures of 800–900 °C. There was a significant increase in the residual strength of 38–42% for *f*_0.2*p*_ and 70–80% for *f_u_*, and this was accompanied by a more sudden and less ductile failure. This occurred because the bars were effectively annealed; there was a change in phase, and then the annealing occurred through quenching in water. This effect did not occur for the stainless steel rebars, which experienced a less significant change in phase and only a variation in the grain texture due to cold working. The total strain at failure *ε_f_* presented a loss of 84% at 800 °C and 79% at 900 °C against the virgin sample. This was caused by the carbon steel rebar being heated above the phase transformation temperature of 737 °C and then being rapidly cooled, inducing the transformation of martensite and resulting in a stronger but more brittle rebar [9].

The air-cooled and slow-cooled samples exposed to temperatures of 800 °C and above presented a decline in strength and increase in strain, similar to the austenitic stainless steel data sets. The residual *f*_0.2*p*_ and *f_u_* values were reduced by 26–47% and 16–28%, respectively, when compared with the virgin sample. The residual strain values presented the greatest rise at 96–135% for *ε_u_* and 74–107% for *ε_f_* against the virgin sample.

**Figure 11:**
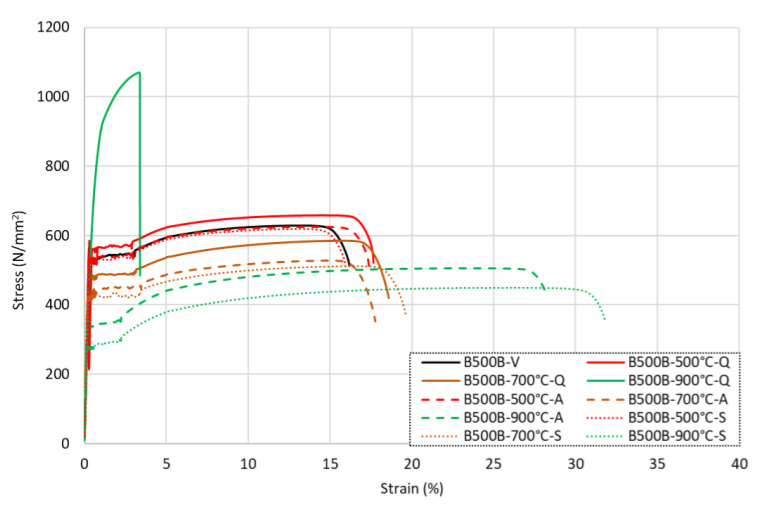
Stress–strain response for grade B500B carbon steel rebars following exposure to 500 °C, 700 °C, and 900 °C, followed by cooling.

Figure 11 presents the carbon steel rebars that were heated to 500 °C, 700 °C, and 900 °C and then cooled in the three different regimes to directly assess the influence of the cooling rate on the residual properties. As stated before, there was clearly a quite notable difference in the response for B500B-800-Q and B500B-900-Q, owing to the successful transformation to the martensite phase. For the other samples, in all cases, the quenched samples which were cooled the quickest in this test series had the greatest residual strength, regardless of the temperature that they were exposed to. This was followed by the air-cooled samples, whilst the slow-cooled samples had the lowest residual strengths. The residual strain increase for the slow-cooled samples was constantly greater than the air-cooled samples.

## 4. Results from the Metallurgical Investigation

In addition to the mechanical tests previously described, a series of metallurgical tests was performed for grade 1.4301. Grades 1.4401 and 1.4436 were not included in the metallurgical testing, as they did not display significantly different behaviour during the mechanical testing. The metallurgical investigation was undertaken on the axial face of the rebar offcuts taken from the same manufacturing batch as the rebar used for tensile testing. The results in this section are given in terms of diffractograms, which present the intensity of the phase identification (intensity, which is a dimensionless measure) against the angle of detection (2theta). These were produced through X-ray diffraction for all samples, which were heated to target temperatures between 100 and 900 °C and then cooled using the three different methods described before (i.e., quenching in water, air-cooling, and slow-cooling in the furnace). The positions of both the austenite and martensite phase peaks are indicated in the diagrams and are labelled with either a solid dot or empty circle for the austenite and martensite peaks, respectively. Notably, only 2theta between 40 and 60° is presented, as this is the range of most significance for austenite and martensite microstructure transformation.

### Grade 1.4301

**Figure 12:**
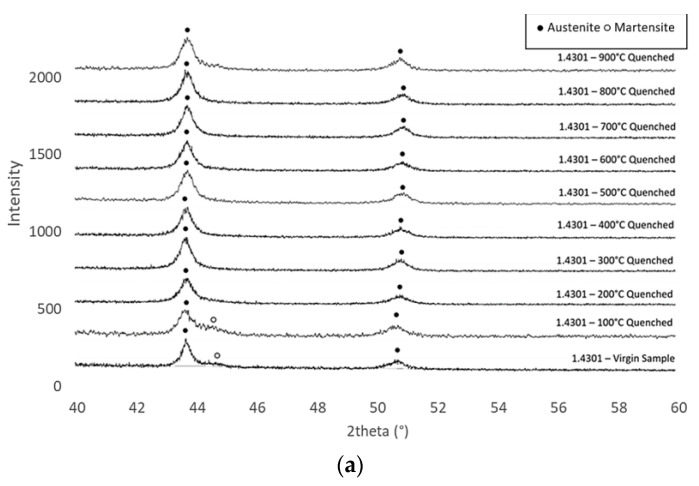

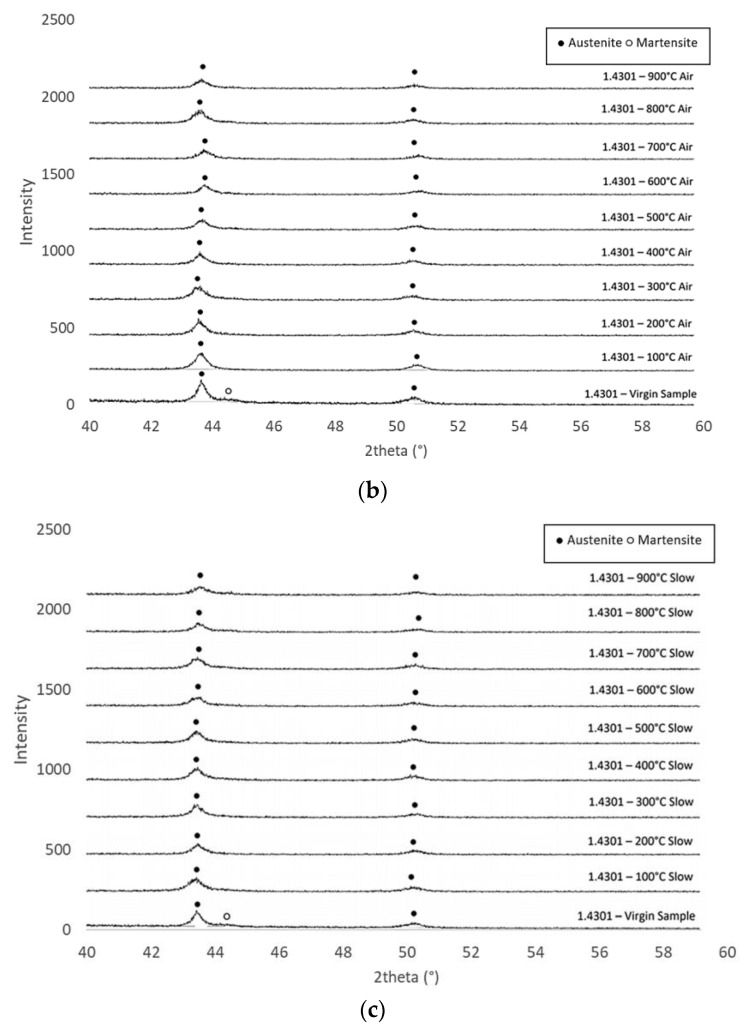
Data from the diffractograms for grade 1.4301 reinforcing bars that were heated to various temperatures as indicated and then cooled (**a**) quickly by quenching in cold water, (**b**) naturally in air, and (**c**) slowly in the furnace.

The diffractograms for the grade 1.4301 samples are shown in Figure 12 for the bars that were (a) quenched in water, (b) air-cooled, and (c) slow-cooled. Each diffractogram is compared against the unheated virgin sample. For the virgin sample, there was a small presence of martensite immediately after the austenite peak at 43.8°, and this was a relatively minor phase identified by the steady decline following the first peak, as opposed to the sharper and more uniform decline found at higher temperatures. It is noteworthy that martensite makes the material stronger and harder compared with alloys without martensite. With reference to Figure 12a, this same pattern, as demonstrated for the virgin sample, was replicated for the sample that was exposed to 100 °C before quenching in water, as there was a small presence of martensite after the initial austenite phase between 43.5 and 44°. For the rest of the quenched samples which were heated to 200 °C and above, as well as all of the samples that were air-cooled or slow-cooled as shown in Figure 12b,c, there was no martensite phase visible in the diffractograms, and there was a relatively smooth decline in terms of intensity visible from the first austenite peak.

**Figure 13:**
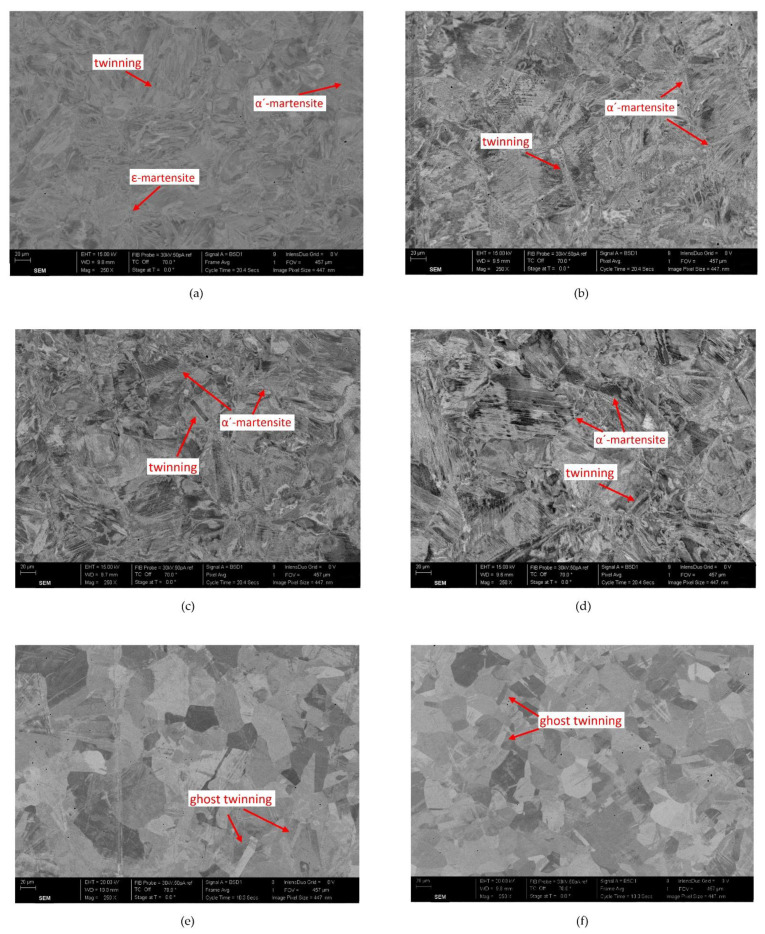

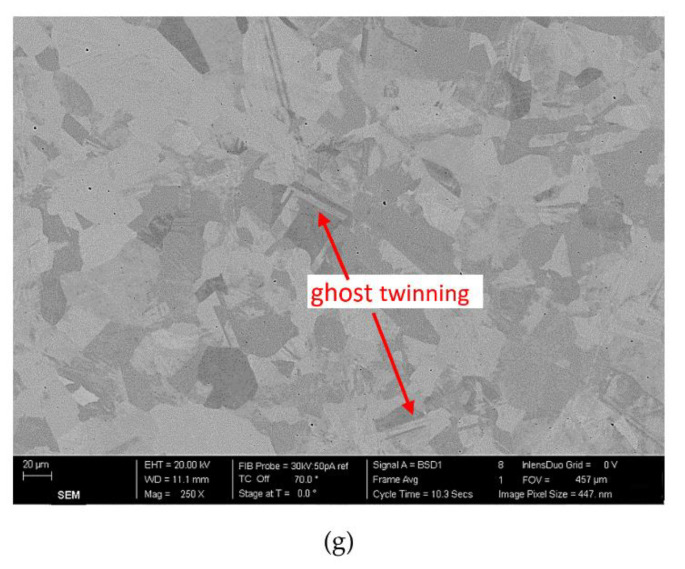
The grain imagery for grade 1.4301 reinforcing bars taken at 250× magnification, including (**a**) the virgin sample, (**b**) the bar exposed to 500 °C and subsequently cooled by quenching in water, (**c**) the bar exposed to 500 °C and subsequently cooled by air cooling, (**d**) the bar exposed to 500 °C and subsequently cooled by slow cooling, (**e**) the bar exposed to 900 °C and subsequently cooled by quenching in water, (**f**) the bar exposed to 900 °C and subsequently cooled by air cooling, and (**g**) the bar exposed to 900 °C and subsequently cooled by slow cooling.

For the grain inspection, the complete microscopic imagery for grade 1.4301 is presented in Figure 13. From a visual inspection, the size, shape, and dispersion of the grain samples, as well as a change in the strengthening mechanisms, were readily observed for (a) the virgin sample, (b–d) the samples heated to 500 °C and then cooled, and (e,f) those that were heated to 900 °C before cooling. The virgin sample shown in Figure 13a had the strengthening mechanisms produced through the cold-working labelled on this image (i.e., α´-martensite, ε-martensite, and twinning). Following exposure to 500 °C and subsequent cooling by all three cooling methods, the strengthening mechanisms became more defined, as shown in Figure 13b–d. This explains the 13–15% rise in *f*_0.2*p*_ and 4–5% rise in *f_u_* that were presented and discussed earlier in this paper for the specimens that were heated in this temperature range.

Theoretically, once the stainless steel is exposed to temperatures exceeding 727 °C, the strengthened alloy begins to actively revert to γ-austenite through the formation of new γ-austenite grains within the grain boundaries of the α′-martensite grains, and following exposure to 900 °C, a complete transformation to γ-austenite is achieved, with some ghost twinning remaining visible. This is presented in Figure 13e–g, where for the samples that were exposed to temperatures of 900 °C before cooling (by any cooling method), the grains reverted to an absolute γ-austenite alloy.

A comparison of the mean grain diameter of the samples exposed to 900 °C and subsequently cooled was also undertaken to understand the influence of the grain size on the material. The quenched sample, as shown in Figure 13e, presented the largest mean grain diameter with a size of 42 μm, whilst the air-cooled sample (Figure 13f) presented a mean grain diameter of 36 μm, and the slow-cooled sample (Figure 13g) had the smallest grains, with a mean grain diameter of 35 μm. Despite a variance in grain size, no significant response in terms of the material strength was noted. On the other hand, in terms of strain, the *ε_f_* values presented earlier in this paper for the grade 1.4301 rebar that was exposed to 900 °C and then quenched showed the greatest increase of those examined in this study, as it was 272% of the corresponding virgin sample value. The equivalent increase for the air-cooled and slow-cooled bars was 259% and 256%, respectively. This indicates that the larger the grain size, the greater the total strain at failure.

## 5. Discussion

For austenitic stainless steel-reinforced concrete structures to remain viable for reuse following a fire, the residual mechanical values of the reinforcement bars would need to meet the requirements given in Part 7 of BS 4449 (2016). All three cooling methods studied in this investigation provided no significant variation in the results based on the cooling rate. The mechanical behaviour for the three studied austenitic grades (i.e., grades 1.4301, 1.4401, and 1.4436) can be broken down into three stages based on the level of temperature exposure, and these are discussed hereafter.

Following relatively low levels of temperature exposure of up to 500 °C, all three materials showed no significant loss in the proof *f*_0.2*p*_ or ultimate tensile stress *f_u_* but may experience some reduction in the failure strain *ε_f_* compared with the unheated virgin sample. This is because following exposure to temperatures of up to 500 °C, the γ-austenite grains cannot form in the alloy, whilst any strengthening or brittleness is the result of the ε-martensite or twins completing the transformation to α´-martensite. For grades 1.4401 and 1.4436, the addition of molybdenum in the alloy makes these materials more resistant to change following exposure to temperatures of up to 500 °C, and there are insignificant changes to the microstructure. This is further supported by the measured mechanical properties (i.e., *f*_0.2*p*_, *f_u_*, *ε_f_*, and *ε_u_*), which remained within 10% of their corresponding virgin sample values, whilst the grade 1.4301 samples had a significant increase in strength and a reduction in ductility.

When exposed to moderate temperatures of 600–700 °C, regardless of the cooling rate, all of the samples showed an increase in strength compared with the virgin samples, although this was lower than for the samples that were heated to 500 °C before cooling. They also had notable changes in the strain behaviour. For example, following exposure to 600 °C and subsequent cooling, grades 1.4301 and 1.4436 had a disproportionately high increase in *ε_u_* relative to *ε_f_*. For 1.4301, the increase in *ε_u_* was 56–67%, whilst *ε_f_* increased by between 3 and 10% compared with the virgin sample. The grade 1.4436 samples had an increase of 61–72% for *ε_u_* and an increase of 11–24% for *ε_f_* compared with the corresponding virgin values. Grade 1.4401 presented more comparable increases in *ε_u_* and *ε_f_*, being equal to 19–25% and 13–17%, respectively. The reason for this higher increase in *ε_u_* for different grades is largely owing to the level of cold-working which was performed on the rebars during production. In the test programme, the rebars began to lose their mechanically-induced cold-working properties following exposure to higher levels of elevated temperatures, thus resulting in a more curved stress–strain response. This same pattern of behaviour was also observed for the samples that were heated to 700 °C. Overall, the grade 1.4301 rebars retained more of their mechanical properties compared with the molybdenum-containing grades 1.4401 and 1.4436.

For the samples exposed to 800 °C temperatures, all of the grades showed different levels of reduction in strength, accompanied by an increase in ductility. This was because the stainless steels had begun to actively undergo recrystallisation but failed to do so completely due to a shortage in either the temperature or time of exposure. On the other hand, for the samples heated to 900 °C, the requirements for active recrystallisation were achieved, resulting in a complete γ-austenite dominant microstructure.

With reference to the influence of the cooling method and therefore the cooling rate, on the retention of strength and ductility for austenitic stainless steel reinforcement, as previously stated, this parameter did not appear to have a major influence on the mechanical response. This observation was clear from the results of the mechanical tests and was also verified from the material characterization and microscopic imagery, as identical results were observed for the three cooling methods. In addition, each test scenario was repeated three times for the different cooling methods with very similar behaviour observed, thus providing further assurance of the reliability of the data.

Finally, regarding the modulus of elasticity *E*, it is noteworthy that there was very little change observed for any of the specimens examined in this study. This is to be expected, as for all stainless steels, regardless of the heat treatment or level of mechanical work, the modulus of elasticity should remain near that of its parent element (i.e., iron) at 200 GPa [10]. The slight variation in elastic modulus values presented herein is attributed to the testing process and laboratory limitations, as also described by other researchers (e.g., Lord and Morell, 2010 [11] and Chen et al., 2016 [12]).

## 6. Conclusions

This paper presents the details, results, and analysis from a series of tests on austenitic stainless steel reinforcement following exposure to fire, followed by cooling. This information was previously not available, and therefore it was difficult for engineers to make informed decisions on the structural integrity of stainless steel reinforced concrete structures following a fire. In this paper, consideration was given to the material properties of three different grades of austenitic stainless steel rebar following exposure to varying degrees of elevated temperatures, followed by cooling. A total of 204 samples were examined in the programme, including 120 mechanical tests and 84 metallurgical tests. The key observations and conclusions from this study are summarised as follows:

(1) All of the stainless steel reinforcing bars examined in this programme retained the majority of their mechanical properties, even following exposure to high levels of elevated temperature of up to 700 °C.
(2) The cooling rate was not shown to have a significant influence on the retention of the mechanical properties for the range of rates examined herein.
(3) More specifically, following exposure to the lower examined temperature range of between 100 °C and 500 °C, in all circumstances, the rebar remained useable and retained all of its strength and ductility following cooling. At the upper limit of 500 °C, the rebars presented an increase in strength, with the grade 1.4301 alloy presenting an *f*_0.2*p*_ increase of up to 15%. The increase in strength was due to the enhanced transformation of α′-martensite from the incomplete ε-martensite and the twins produced through cold-working.
(4) With exposure to the moderate temperature range of 600 °C and 700 °C, whilst the alloys retained enough strength to remain usable, they showed a characteristic change in the shape of the stress–strain curve. The samples presented a more rounded response and elongated further due to gradual loss of the cold-worked effect on the materials such as the twins, ε-martensite, and α′-martensite.
(5) Once the rebar was exposed to very high temperatures of 800–900 °C, in some scenarios, enough strength was retained such that the rebar remained usable. However, due to the excessive elongation as well as the strength loss percentage, at this stage, the rebar had undergone active recrystallisation, recrystalising partially to γ-austenite at 800 °C and completely by 900 °C. As the retained strength was a product of the degree of initial cold-working during manufacturing, and this is not openly accessible information, once the rebar has been exposed to temperatures of 800 °C and 900 °C, it should not be considered fit for use.
(6) Although this paper was focussed on the mechanical properties of stainless steel reinforcement under static loading conditions, it is recommended that future work should include an analysis of the material hardness and fracture toughness, as well as a study into the properties under cyclic loading.

## Figures and Tables

**Table 1:** Previous research into the behaviour of stainless steel reinforcement.

		Heating			Cooling	
Source	Grade	Rate (°C/min)	Soaking Time	Target Temperature (°C)	Rate (°C/min)	Cooling Method
Felicetti et al. [2]	1.4307 (cold-worked and hot-rolled)	3	60 mins	20, 200, 400, 550, 700, 850	3	Controlled cooling in furnace
Zeng et al. [3]	1.4401	n/a	60 min, 5 h, 10 h, 24 h, 48 h, 1 week	600, 700, 800, 900	n/a	Annealed

**Table 2:** Composition of the tested austenitic stainless steels.

	Grade	1.4301			1.4401			1.4436		
Element		Actual	Min	Max	Actual	Min	Max	Actual	Min	Max
%C: Carbon		0.032	-	0.07	0.023	-	0.07	0.028	-	0.05
%Mn: Manganese		1.72	-	2	1.438	-	2	1.36	-	2
%Si: Silicon		0.46	-	1	0.366	-	1	0.36	-	1
%S: Sulphur		0.004	-	0.03	0.027	-	0.03	0.007	-	0.03
%P: Phosphorus		0.039	-	0.045	-	-	0.045	0.031	-	0.045
%Ni: Nickle		8.1	8	10.5	10.54	10	13	10.54	10.5	13
%Cr: Chromium		18.4	17.5	19.5	16.685	16.5	18.5	16.67	16.5	18.5
%Mo: Molybdenum		0.24	-		2.049	2	2.5	2.53	2.5	3
%N: Nitrogen		0.183	-	0.22	0.046	-	0.1	0.061	-	0.11
%Cu: Copper		-	-	-	0.317	-	-	-	-	-
%Ti: Titanium		-	-	-	0.005	-	-	-	-	-

**Table 3:** Post-fire mechanical properties of grade 1.4301 stainless steel reinforcing bar.

	1.4301				
Specimen	*E*	*f*_0.2*p*_	*f_u_*	*ε_u_*	*ε_f_*
	(GPa)	(N/mm^2^)	(N/mm^2^)	(%)	(%)
Virgin	192.3	727.3	910.7	9.7	23.5
100 °C-Q	183.5	758.0	915.5	9.6	24.8
200 °C-Q	189.0	784.8	934.9	9.6	19.9
300 °C-Q	185.3	799.6	920.5	9.7	19.9
400 °C-Q	183.4	813.7	934.1	9.6	19.1
500 °C-Q	191.5	834.4	953.8	9.7	19.3
600 °C-Q	195.1	792.3	943.5	16.2	22.6
700 °C-Q	194.1	720.0	900.6	22.0	28.7
800 °C-Q	181.9	461.9	773.1	38.0	44.1
900 °C-Q	181.9	320.1	716.0	56.7	63.5
100 °C-A	190.2	744.9	922.8	9.7	21.8
200 °C-A	186.8	750.2	911.8	9.6	22.1
300 °C-A	191.1	808.9	938.9	9.7	18.7
400 °C-A	192.8	839.4	949.3	9.6	20.0
500 °C-A	196.7	821.0	945.6	9.7	21.5
600 °C-A	198.3	806.3	935.6	15.2	22.0
700 °C-A	192.8	751.6	907.7	20.6	27.2
800 °C-A	192.1	544.8	800.8	29.7	35.9
900 °C-A	197.7	338.5	704.4	54.4	60.5
100 °C-S	182.1	730.1	905.1	9.6	21.5
200 °C-S	187.7	779.6	918.1	9.6	18.8
300 °C-S	184.4	808.9	929.5	9.6	20.2
400 °C-S	186.5	812.9	934.7	9.7	21.3
500 °C-S	194.9	831.6	957.7	9.6	19.2
600 °C-S	192.2	794.0	929.5	15.5	20.9
700 °C-S	196.9	755.8	915.4	18.4	23.1
800 °C-S	195.3	489.0	783.2	33.0	39.3
900 °C-S	195.0	336.1	704.6	52.6	59.7

**Table 4:** Post-fire mechanical properties of grade 1.4401 stainless steel reinforcing bar.

	1.4401				
Specimen	*E*	*f*_0.2*p*_	*f_u_*	*ε_u_*	*ε_f_*
	(GPa)	(N/mm^2^)	(N/mm^2^)	(%)	(%)
Virgin	191.6	604.0	723.6	14.3	20.0
100 °C-Q	198.2	579.8	711.6	15.2	21.5
200 °C-Q	195.5	580.4	717.2	14.2	19.4
300 °C-Q	184.5	610.7	721.9	13.6	20.5
400 °C-Q	183.8	637.3	743.0	13.7	20.2
500 °C-Q	189.7	624.4	746.5	13.1	17.8
600 °C-Q	187.4	569.5	715.9	17.9	22.7
700 °C-Q	194.4	498.6	685.8	25.5	31.5
800 °C-Q	187.2	424.7	649.7	32.1	38.9
900 °C-Q	185.5	308.5	577.7	47.5	53.7
100 °C-A	187.6	583.3	702.0	14.4	20.4
200 °C-A	183.6	586.1	711.2	13.9	19.0
300 °C-A	187.6	619.0	723.9	13.6	20.5
400 °C-A	180.6	619.5	724.7	13.6	21.6
500 °C-A	184.7	606.9	722.4	13.6	18.3
600 °C-A	188.3	581.8	721.5	17.0	22.5
700 °C-A	185.1	527.7	687.7	22.0	28.0
800 °C-A	181.3	464.2	663.2	30.2	35.9
900 °C-A	185.4	309.1	586.2	45.5	51.2
100 °C-S	182.9	641.1	707.0	14.5	20.6
200 °C-S	187.1	613.9	718.0	12.8	18.4
300 °C-S	188.2	610.8	720.5	13.0	18.3
400 °C-S	184.3	632.0	728.5	13.6	21.6
500 °C-S	188.7	630.0	741.7	13.3	20.2
600 °C-S	180.0	594.3	730.1	17.9	23.3
700 °C-S	196.4	542.1	705.2	24.0	29.6
800 °C-S	191.7	466.8	663.2	29.3	34.9
900 °C-S	184.3	297.4	589.2	46.6	53.4

**Table 5:** Post-fire mechanical properties of grade 1.4436 stainless steel reinforcing bar.

	1.4436				
Specimen	*E*	*f*_0.2*p*_	*f_u_*	*ε_u_*	*ε_f_*
	(GPa)	(N/mm^2^)	(N/mm^2^)	(%)	(%)
Virgin	186.7	672.2	805.3	9.7	19.6
100 °C-Q	181.7	678.0	794.4	9.9	19.9
200 °C-Q	191.4	693.8	805.0	9.7	17.7
300 °C-Q	185.9	720.8	822.6	9.7	19.9
400 °C-Q	188.4	726.0	831.7	9.7	21.0
500 °C-Q	186.3	728.0	842.3	9.7	17.7
600 °C-Q	184.0	689.3	828.4	17.1	24.3
700 °C-Q	192.6	601.0	778.2	19.7	24.5
800 °C-Q	184.4	500.5	726.2	29.7	35.4
900 °C-Q	196.0	261.7	617.6	56.0	61.8
100 °C-A	180.2	664.4	790.9	9.7	19.6
200 °C-A	184.3	682.0	795.9	9.7	19.5
300 °C-A	193.5	717.2	815.9	9.7	18.1
400 °C-A	182.0	731.1	825.8	9.8	17.2
500 °C-A	185.8	745.4	846.0	9.9	15.8
600 °C-A	189.2	710.2	831.1	15.5	21.8
700 °C-A	181.9	620.8	781.5	22.0	28.0
800 °C-A	190.0	529.9	728.3	29.6	35.2
900 °C-A	190.5	262.5	614.9	55.7	61.7
100 °C-S	192.3	664.7	789.2	9.7	19.6
200 °C-S	187.2	684.5	791.6	9.7	18.1
300 °C-S	182.1	723.2	813.5	9.7	16.8
400 °C-S	184.8	727.2	821.8	9.6	18.4
500 °C-S	189.2	735.4	841.5	9.6	21.6
600 °C-S	184.9	687.5	816.4	16.8	22.3
700 °C-S	181.6	622.7	777.7	21.6	27.5
800 °C-S	189.5	522.9	725.9	30.0	35.6
900 °C-S	188.8	265.1	621.0	59.4	67.2

**Table 6:** Post-fire properties of carbon steel grade B500B reinforcing bar.

	B500B				
Specimen	*E*	*f*_0.2*p*_	*f_u_*	*ε_u_*	*ε_f_*
	(GPa)	(N/mm^2^)	(N/mm^2^)	(%)	(%)
Virgin	212.0	530.9	628.1	11.7	16.2
100 °C-Q	201.2	533.2	621.8	10.5	12.5
200 °C-Q	198.8	520.3	613.5	11.4	13.1
300 °C-Q	197.1	513.2	615.3	10.7	15.0
400 °C-Q	199.5	548.3	642.3	11.8	16.1
500 °C-Q	194.6	559.4	657.9	13.1	17.7
600 °C-Q	220.0	565.8	648.6	13.7	18.5
700 °C-Q	209.5	479.5	583.9	13.9	18.6
800 °C-Q	186.8	731.9	1130.3	1.9	2.6
900 °C-Q	191.6	756.2	1070.1	2.7	3.4
100 °C-A	198.5	527.2	620.1	12.2	16.5
200 °C-A	218.6	531.3	611.4	12.0	13.8
300 °C-A	199.2	532.4	628.7	14.1	19.1
400 °C-A	217.4	533.1	625.3	11.2	15.5
500 °C-A	203.3	533.0	624.9	12.7	17.4
600 °C-A	197.0	526.4	611.6	14.7	19.4
700 °C-A	202.5	438.2	526.3	13.1	17.8
800 °C-A	201.0	392.2	528.6	24.5	30.9
900 °C-A	215.5	336.7	505.7	23.0	28.2
100 °C-S	209.1	536.0	628.1	13.0	17.5
200 °C-S	201.7	532.2	618.5	11.5	13.4
300 °C-S	204.7	535.4	628.6	11.5	15.8
400 °C-S	199.5	522.8	618.1	10.8	13.3
500 °C-S	205.2	523.9	618.2	11.6	16.0
600 °C-S	197.0	524.7	600.6	15.5	20.4
700 °C-S	192.9	423.6	510.9	14.9	19.6
800 °C-S	198.6	327.7	468.4	27.6	33.6
900 °C-S	197.2	280.7	449.5	25.5	31.8

## Glossary

Key	
*f_u_*	Ultimate tensile stress
*f*_0.2*p*_	0.2% proof stress
*ε_f_*	Total strain at failure
*ε_u_*	Percentage total elongation at maximum force
*E*	Modulus of elasticity
